# Sample Size Considerations for Fine-Tuning Large Language Models for Named Entity Recognition Tasks: Methodological Study

**DOI:** 10.2196/52095

**Published:** 2024-05-16

**Authors:** Zoltan P Majdik, S Scott Graham, Jade C Shiva Edward, Sabrina N Rodriguez, Martha S Karnes, Jared T Jensen, Joshua B Barbour, Justin F Rousseau

**Affiliations:** 1 Department of Communication North Dakota State University Fargo, ND United States; 2 Department of Rhetoric & Writing The University of Texas at Austin Austin, TX United States; 3 Department of Neurology The Dell Medical School The University of Texas at Austin Austin, TX United States; 4 Department of Rhetoric & Writing University of Arkansas Little Rock Little Rock, AR United States; 5 Department of Communication The University of Illinois at Urbana-Champaign Urbana, IL United States; 6 Statistical Planning and Analysis Section Department of Neurology The University of Texas Southwestern Medical Center Dallas, TX United States; 7 Peter O’Donnell Jr. Brain Institute The University of Texas Southwestern Medical Center Dallas, TX United States

**Keywords:** named-entity recognition, large language models, fine-tuning, transfer learning, expert annotation, annotation, sample size, sample, language model, machine learning, natural language processing, disclosure, disclosures, statement, statements, conflict of interest

## Abstract

**Background:**

Large language models (LLMs) have the potential to support promising new applications in health informatics. However, practical data on sample size considerations for fine-tuning LLMs to perform specific tasks in biomedical and health policy contexts are lacking.

**Objective:**

This study aims to evaluate sample size and sample selection techniques for fine-tuning LLMs to support improved named entity recognition (NER) for a custom data set of conflicts of interest disclosure statements.

**Methods:**

A random sample of 200 disclosure statements was prepared for annotation. All “PERSON” and “ORG” entities were identified by each of the 2 raters, and once appropriate agreement was established, the annotators independently annotated an additional 290 disclosure statements. From the 490 annotated documents, 2500 stratified random samples in different size ranges were drawn. The 2500 training set subsamples were used to fine-tune a selection of language models across 2 model architectures (Bidirectional Encoder Representations from Transformers [BERT] and Generative Pre-trained Transformer [GPT]) for improved NER, and multiple regression was used to assess the relationship between sample size (sentences), entity density (entities per sentence [EPS]), and trained model performance (*F*_1_-score). Additionally, single-predictor threshold regression models were used to evaluate the possibility of diminishing marginal returns from increased sample size or entity density.

**Results:**

Fine-tuned models ranged in topline NER performance from *F*_1_-score=0.79 to *F*_1_-score=0.96 across architectures. Two-predictor multiple linear regression models were statistically significant with multiple *R*^2^ ranging from 0.6057 to 0.7896 (all *P*<.001). EPS and the number of sentences were significant predictors of *F*_1_-scores in all cases ( *P*<.001), except for the GPT-2_large model, where EPS was not a significant predictor (*P*=.184). Model thresholds indicate points of diminishing marginal return from increased training data set sample size measured by the number of sentences, with point estimates ranging from 439 sentences for RoBERTa_large to 527 sentences for GPT-2_large. Likewise, the threshold regression models indicate a diminishing marginal return for EPS with point estimates between 1.36 and 1.38.

**Conclusions:**

Relatively modest sample sizes can be used to fine-tune LLMs for NER tasks applied to biomedical text, and training data entity density should representatively approximate entity density in production data. Training data quality and a model architecture’s intended use (text generation vs text processing or classification) may be as, or more, important as training data volume and model parameter size.

## Introduction

### Background

Named entity recognition (NER) has many applications in biomedical and clinical natural language processing (NLP). As its core function, NER identifies and categorizes specific terms or phrases representing people, places, organizations, and other entities. It has been used to identify or extract named entities in free-text clinical notes and reports in the secondary analysis of electronic health records [1,2]. NER has also been used alone or as part of an NLP pipeline to detect protected health information in order to deidentify clinical text for secondary analysis [3,4]. Additionally, NER has been used to identify and classify medications [5,6], specific disease and clinical condition entities [7], and laboratory tests [8] into existing taxonomies for purposes of secondary research, cohort generation, or clinical decision support [9-12]. While NER solutions have a long history of applications in NLP and clinical NLP domains, their effectiveness has recently been enhanced through the addition of large language models (LLMs) in relevant data parsing pipelines. LLMs have become an integral part of research pipelines in fields as diverse as digital humanities [13], computational social science [14], bioinformatics, applied ethics, and finance.

LLMs, such as GPT-3, have demonstrated remarkable performance across a variety of tasks. For instance, the GPT-3.5–powered LLM application ChatGPT performed close to or at the passing threshold of 60% accuracy on the United States Medical Licensing Exam (USMLE) without the specialized input of human trainers [15]. Widely available models, such as Google’s Bidirectional Encoder Representations from Transformers (BERT) or OpenAI’s Generative Pre-trained Transformer (GPT) series, are trained, bidirectionally or unidirectionally, on large volumes of generic textual data, designed to represent a wide array of common language use contexts and scenarios [16]. In specialized use contexts, these generic models often fail to accurately classify information because the language structures that require classification—their words, syntax, semantic context, and other textual or lexical signatures—are sparsely represented in the data that were used to train the generic model [17,18]. Some language models, such as ElutherAI’s GPT-J-6B, are trained on open-source language modeling data sets curated from a mix of smaller open web crawl data sets alongside more technical papers from PubMedCentral and arXiv and can offer improved classification accuracy for technical applications [19]. Nevertheless, specialized tasks often require fine-tuning of general-purpose LLMs. Fine-tuning provides a way of overcoming the limitations of generic LLMs by augmenting their training data with data selected to more accurately reflect the target domains toward which a model is fine-tuned. The fine-tuning process updates the model’s parameters—the weights that affect which connections between the nodes and layers of a neural network become activated—and so helps a model permanently learn. Unlike practices, such as prompt engineering, that leave the underlying language model untouched, fine-tuning changes the model itself, yielding a new model optimized for the specific use case.

However, fine-tuning LLMs to perform technical, specialized tasks is expensive, because the target domain of a fine-tuned model is usually complex and technical—otherwise, fine-tuning would not be necessary—and it requires annotators with some degree of domain-level expertise, which comes with potentially significant financial and time costs. Indeed, one study of NER annotation speed found it can take between 10 and 30 seconds per sentence for experts to annotate named entities [8]. The gold-standard annotated BioSemantics corpus is composed of 163,219 sentences, which implies an optimal annotation time of over 11 weeks at 40 hours per week (453.39 h) [20]. This estimate, of course, excludes the time required for annotator training and interannotator reliability assessments, and because fine-tuning adjusts many or all of the model’s parameters, it consumes computational resources. Time and power consumption for fine-tuning scales with training data size [21,22] and with the size of the underlying model that is computed. As of the date of writing, for example, it would be unrealistic to fine-tune very large models such as GPT-4.

These limitations notwithstanding, it is increasingly recognized that long-standing presumptions about sufficiently large training data sets are likely substantially inflated [23]. We suspect this comes from a research and development environment dominated by a significant focus on promulgating new models that can claim to be state-of-the-art (SOTA) based on some preidentified benchmark. In a research environment dominated by so-called “SOTA chasing,” ever larger data sets are often required to eke out minor performance improvements over the previous benchmarks. Notably, development teams from disciplines with generally small research budgets have found that fine-tuning can result in substantial performance improvements from relatively small amounts of expert-annotated data [13,24] or from a combination of prelearning and transfer learning followed by a brief fine-tuning phase [25]. In one case, significant improvements over the baseline were derived from training samples as small as 50 lemmas [13]. Despite the growing recognition that smaller gold-standard training sets can provide substantial performance improvements, there is little in the way of actionable guidance for sample size and sample curation.

The primary goal of this study is to establish some initial baselines for sample size considerations in terms of training set size and relevant entity density for NER applications in specialized technical domains. To that end, we have conducted a fine-tuning experiment that compares the performance improvements resulting from 2500 randomly selected training data sets stratified by size. These training sets were used to fine-tune 4 distinct language models to perform NER in a highly specific language domain: the identification of 2 internal components (conflict sources and conflict targets) in conflicts of interest (COI) disclosures. The results presented below indicate that only relatively small samples are required for substantial improvement. They also demonstrate a rapidly diminishing marginal return for larger sample sizes. In other words, while larger and larger sample sizes may be useful for “SOTA chasing,” their value for fine-tuning LLMs shrinks beyond a certain threshold, which we estimate below. These findings provide actionable guidance about how to select and generate fine-tuning samples by attending to issues of relevant token density. As such, they should have great value for NER applications that rely on them.

### Literature Review

During our initial review of the literature, we were unable to locate any widely accepted, evidence-based guidance on appropriate sample sizes for training data in NER fine-tuning experiments. Therefore, to evaluate the state of the field, we conducted a literature search focused on identifying existing practices. We searched PubMed for prior relevant work to determine current sample size conventions in NER fine-tuning. We used a simple search strategy “(“named entity recognition” OR “entity extraction”) AND (fine tuning OR transfer learning) AND (annotat*),” which returned 138 relevant papers. We reviewed each of these papers and extracted information related to human-annotated NER training sets. Specifically, for each paper, we assessed if a human-annotated training set was used, and if so, we extracted data on sample units, sample size, and any available sample size justification. In cases where authors described the size of human-annotated training sets on multiple levels (eg, number of documents, number of sentences, and number of entities), we prioritized units that would most effectively guide prospective sampling. This emphasis meant that we prioritized sentences (as they are comparable across document types and identifiable without annotation) over documents (which vary widely in length) or entities (which cannot be assessed until after annotation). In cases where multiple human-annotated samples were used, we noted the largest reported sample as indicative of the researchers’ sense of the sample necessary to conduct the research in its entirety. Additionally, for each paper that made use of a human-annotated training set, we sought to identify any possible justifications for the chosen sample size. We anticipated that common justifications might include (1) collecting a sample sufficient to achieve target performance, (2) collecting a sample consistent with or larger than prior work, or (3) collecting a sample appropriate given relevant power calculations.

Of the papers surveyed, the majority (93/138, 67.4%) reported the use of human-annotated NER training data. The remaining (45/138, 32.6%) papers used only computational approaches to curate training data sets. Notably, many papers reported using a mix of human-annotated and computationally-annotated training sets or performing multiple experiments with different training sets. As long as any given paper used at least 1 human-annotated training set, it was included in the tally. Reported sample units varied quite widely across papers with many reporting only the number of documents used. Document types were similarly variable and specific to research contexts. For example, several papers reported training sample sizes as the number of clinical notes, number of published abstracts, or number of scraped tweets. In contrast, some papers reported sample size using non-context-specific measures such as sentences, entities, or tokens. Given this variety, we classified sample units as belonging to 1 of 6 common categories: clinical notes or reports, sentences, abstracts or papers, entities, tokens, or others. The most commonly used sample unit was clinical notes or reports (34/93, 37%) followed by sentences and papers or abstracts (21/93, 23%). Sample size ranges also varied widely by unit type, as would be expected. The smallest clinical notes or reports sample used a scant 17 documents [26], but this was likely a larger sample than the smallest reported sentence sample size of 100 [27]. Among the papers reporting nondocument type specific sample units, human-annotated data sets ranged from 1840 tokens to 79,401 tokens (mean 42,121 tokens); from 100 entities to 39,876 entities (mean 15,957 entities); and from 100 sentences to 360,938 sentences (mean 26,678 sentences). Details on the sample size range by sample type are available in Table 1. Complete details on each paper’s approach to sample size are available in Multimedia Appendix 1.

Of the 93 papers that used human-annotated NER training data, only 3 (3%) papers provided an explicit justification for the chosen sample size. In each case, the justification for the sample size was based on a reference to prior relevant work and determined to be as large or larger than a sample used in the previously published work [28-30]. Ultimately, the wide range of sample reporting practices and the broad lack of attention to sample size justification indicate a strong need for explicit sample selection guidance for fine-tuning NER models. This paper contributes to addressing this need.

**Table 1:** Unit types, number of papers by type, and sample size means and ranges.

Unit type	Papers (n=93), n (%)	Sample size, mean	Sample size, range
Clinical notes or reports	34 (37)	709	17-5098
Abstracts or papers	21 (23)	1966	20-7000
Sentences	21 (23)	26,678	100-360,938
Other	9 (10)	5979	47-25,678
Entities	5 (5)	15,957	100-39,876
Tokens	3 (3)	42,121	1840-79,401

## Methods

### Overview

The primary aim of this study was to evaluate sample size considerations for fine-tuning LLMs for domain- and context-specific NER tasks. Specifically, the goal was to evaluate how changes in retraining data set sizes and token density impact overall NER performance. To accomplish this task, we used stratified random samples of training sets to create 2500 fine-tuned instances of RoBERTa_base, GatorTron_base, RoBERTa_large, and GPT-2_large. In what follows, we describe (1) the data and target NER task, (2) the gold-standard annotation protocol, (3) the fine-tuning approach, and (4) our sample feature analysis.

### Data Description and Context

We selected COI disclosures in biomedical literature as a highly domain-specific, technical language context suitable for the goals of this paper. In recent years, significant research efforts have been devoted to studying the effects of financial COI on the biomedical research enterprise [31-33], finding that COI is associated with favorable findings for sponsors [31], increased rates of “spin” in published reports [34], increased likelihood of trial discontinuation or nonpublication [35], editorial and peer reviewer biases [36], and increased adverse events rates for developed products [37]. Unfortunately, as compelling as this body of evidence is, a recent methodological review of research in this area indicates that most studies treat COI as a binary variable (present or absent) rather than quantifying COI rates or disaggregating COI types [32]. This limitation in the available evidence is, no doubt, driven in part by the data structures of COI reporting. When COI are reported, they are generally reported in unstructured or semistructured text. COI disclosure statements can also be quite long, as individual authors frequently receive and report multiple lines of funding from a wide variety of granting agencies and corporate sponsors. Ultimately, the lack of tabular data structures for COI makes it difficult to extract appropriate information [38] such as the sources and recipients of funding, the precise links between COI sources and recipients, or the quantity and degree of COI in a given disclosure statement.

These limitations notwithstanding, there has been some recent research leveraging informatics techniques, including NER, to transform text disclosure statements into tabular data [18,37]. Recently developed systems leverage NER to identify authors and sponsors as “PERSONs” and “ORGs,” respectively. Secondary processing makes use of regular expressions to parse the types of relationships reported between each NER-identified PERSON and ORG. Since NER-tagging in this context is focused on identifying canonical entity types, applying these tools to COI disclosure statements may seem relatively straightforward at the outset. However, variances in reporting formats and the lack of specific training data on relevant entities present a number of challenges. In the first case, author identification is stymied by different journal guidelines for rendering author names. For example, a disclosure statement for Rudolf Virchow might be rendered as “Rudolf Virchow,” “Virchow,” “Dr. Virchow,” or “RLCV.” Likewise, pretrained NER models have not been found to offer high-quality, out-of-the-box performance for pharmaceutical company names [18]. Variations in incorporation type (Inc, LLC, GmbH, etc) typically induce entity boundary issues, and multinational companies often report national entity names (eg, Pfizer India), leading standard NER models to assign inappropriate geopolitical entity tags. Finally, effective NER on COI disclosure statements is also challenged by the atypical distribution of relevant tokens. It is not uncommon for a single sentence in a disclosure to have a dozen author names or a dozen company names, for example, when a disclosure statement lists all authors who have the same COI (eg, “such-and-such authors are employed at MSD”). These atypical sentence structures also occur when a single author has many COIs to disclose, as in, “RLCV receives consulting fees from MSD, Pfizer, GSK, Novartis, and Sanofi.”

To more clearly demonstrate these limitations, we provide the following authentic example from a COI disclosure statement published in a 2018 issue of the *World Journal of Gastrointestinal Oncology* [39]. The following shows the NER tagging performance of RoBERTa_base without fine-tuning:

Sunakawa Y[ORG] has received honoraria from Taiho Pharmaceutical[ORG], Chugai Pharma[ORG], Yakult Honsha[ORG], Takeda[ORG], Merck Serono[ORG], Bayer Yakuhin[ORG], Eli Lilly Japan[ORG], and Sanofi[ORG]; Satake H[ORG] has received honoraria from Bayer[ORG], Chugai Pharma[ORG], Eli Lilly Japan[ORG], Merck Serono[ORG], Takeda[ORG], Taiho Pharmaceutical[ORG] and Yakult Honsha[ORG]; Ichikawa W[ORG] has received honoraria from Chugai Pharma[ORG], Merck Serono[ORG], Takeda Pharmaceutical[ORG], and Taiho Pharmaceutical[ORG]; research funding from Chugai Pharma[ORG], Takeda Pharmaceutical[ORG], and Taiho Pharmaceutical[ORG].

Furthermore, the following shows the NER tags provided by the human annotation team:

Sunakawa Y[PERSON] has received honoraria from Taiho Pharmaceutical[ORG], Chugai Pharma[ORG], Yakult Honsha[ORG], Takeda[ORG], Merck Serono[ORG], Bayer Yakuhin[ORG], Eli Lilly Japan[ORG], and Sanofi[ORG]; Satake H[PERSON] has received honoraria from Bayer[ORG], Chugai Pharma[ORG], Eli Lilly Japan[ORG], Merck Serono[ORG], Takeda[ORG], Taiho Pharmaceutical[ORG] and Yakult Honsha[ORG]; Ichikawa W[PERSON] has received honoraria from Chugai Pharma [ORG], Merck Serono[ORG], Takeda Pharmaceutical[ORG], and Taiho Pharmaceutical[ORG]; research funding from Chugai Pharma[ORG], Takeda Pharmaceutical[ORG], and Taiho Pharmaceutical[ORG].

It is evident that the base LLM classifier makes critical errors that make mapping COI relationships between researchers, funding streams, and funding sources impossible. In the above example, a base-trained classifier mistakenly tags PERSONs as ORGs; elsewhere, we have seen the opposite, where non–fine-tuned classifiers mistakenly identify companies, such as Novartis or Eli Lilly, as PERSONs. General purpose language models (such as BERT and GPT-3) are not well-suited to the NER task of classifying and linking named authors and disclosed payors (pharmaceutical companies, nonprofit foundations, federal funders, etc) because of challenges that arise from the aforementioned lack of standardized disclosure conventions for author names. Likewise, another challenge arises because these models are not well-trained on biomedical companies, nonprofit entities, and federal funders. In this study, as well as earlier research, we found that pharmaceutical companies—frequently named after founding families—are often tagged as PERSONs rather than ORGs. Finally, the linguistic signature of COI disclosure statements is distinctive: COI statements deploy semicolons in nonstandard ways. For large research teams, a single disclosure sentence can cover the length of a long paragraph, and grammatical conventions that govern the relationship between subjects, direct objects, and indirect objects are often elided or circumvented in favor of brevity, which makes linking authors to payors and payors to type of payment challenging. At the same time, the linguistic conventions used for disclosure statements vary between and even within journals, rendering rule-based NER approaches unfeasible. As such, the task of identifying and linking authors to payors and payment types in COI statements is an ideal use case for fine-tuning parameter-dense language models based on gold-standard human annotated COI statements.

### Data Sources and Preprocessing

The data used for fine-tuning COI-relevant NER tags in this study come from COI disclosure statements drawn from 490 papers published in a diverse range of biomedical journals. The selected disclosure statements were randomly sampled from a preexisting data set of 15,374 statements with artificial intelligence–identified COI [40]. The original data set was created by extracting all PubMed-indexed COI statements in 2018. At the time of download, there were 274,246 papers with a COI-statement field in the PubMed XML file. The substantial majority of these are statements of no conflict disclosure, and thus collected statements were analyzed using a custom machine learning–enhanced NER system that can reliably identify relationships between funding entities and named authors [18,37]. The sample used in this study was drawn from the population of COI statements with artificial intelligence–confirmed conflict disclosures.

Two annotators independently tagged named entities in the collected COI statements as either people (PERSON) or organizations (ORG). The PERSON tag was applied to all named authors, regardless of the format of the name. This included initials with and without punctuation, for example, “JAD” or “J.A.D” as well as full names “Jane A. Doe” or names with titles “Dr. Doe.” ORG tags were applied to named pharmaceutical companies, nonprofit organizations, and funding agencies. To ensure that NER tagging was consistent, a random sample of 200 COI statements was tagged by both annotators and assessed for interannotator agreement using interclass correlation coefficient for unit boundaries and Cohen κ for entity type agreement. The raters had 98.3% agreement on unit boundaries (interclass correlation coefficient=0.87, 95% CI 0.864-0.876). For named entities with identical unit boundaries, the classification (PERSON or ORG) agreement was 99.6% (κ=0.989). After this high degree of interrater reliability was established, the annotators independently annotated the remaining COI statements. Prior to training the language model, a third rater reconciled the few annotation disagreements in the initial interrater reliability sample.

### Model Fine-Tuning and Analysis

A subset (147/490, 30%) of the annotated disclosure statements was reserved to serve as an evaluation set. The remaining 343 statements were used to generate 2500 training sets for subsequent experimentation. Each set was created by randomly selecting an N size in 5 preidentified strata of 40 possible sample sizes, at the statement level. The strata included size ranges of 1-40, 41-80, 81-120, 121-160, and 161-200. Once each N size was selected, a random sample of COI statements at that N size was derived. We created 500 random samples within each stratum.

We fine-tuned 4 commonly used language models using the open-source *spaCy* NLP library (version 3.2.1, running on Python version 3.9.7). To ensure the repeatability of results and to make the fine-tuning process as accessible as possible to research teams, we used *spaCy*’s default configuration settings for NER. The selected models included RoBERTa_base, GatorTron_base, RoBERTa_large, and GPT-2_large; for the latter 3, we used the *spacy-transformers* package to access these models through Hugging Face’s *transformers* library. These models were selected to provide a range of parameter sizes (125M to 744M) and to allow for a comparison between language models trained on general use, as well as on biomedical texts specifically. Fine-tuning was performed on *spaCy*’s pretrained transformer pipeline, with only the *transformer* and *NER* pipeline components enabled in the configuration file. All fine-tuning processes were run on a high-performance computing cluster at North Dakota State University’s Center for Computationally Assisted Science and Technology, using AMD EPYC central processing units and NVIDIA graphics processing units. Preprocessing and tokenization were done using *spaCy*’s built-in tokenizer; training runs were optimized with the Adam algorithm, with decay rates of 0.9 (beta1) and 0.999 (beta2) and a learning rate of 0.01. For each training run, *spaCy* was set to check NER classifications against the test set after every 200 iterations within an epoch, to generate language models at regular intervals during the training process, and to stop whenever additional training steps failed to improve the classification metrics. We then extracted the highest-scoring language model from each set, for a total of 2500 fine-tuned language models.

Each of the 2500 retraining sets was subsequently categorized by sample size (measured in the number of sentences) and relevant entity density (entities per sentence [EPS]). Sentence boundaries were determined using the sentencizer in the R *tidytext* (0.3.4) library [41]. Sentences were used to provide a more regularized comparator as disclosure statements vary widely in length. We also focus on sentences as opposed to tokens since the number of sentences in a sample can be identified prospectively (ie, prior to annotation). Multiple regression was used to assess the linear relationship between sample size (number of sentences), entity density (EPS), and trained model *F*_1_-score. Additionally, we used single-predictor threshold regression models for the number of sentences and EPS to evaluate the possibility of diminishing marginal returns from increased sample size or taken density [42]. Threshold regression offers an effective way to model and evaluate nonlinear relationships, and as the term suggests, to identify any threshold effects. Multiple threshold models are available, and our approach relies on a hinge model that can be expressed as follows:



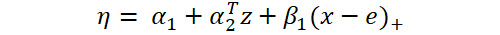



All statistical tests were performed in R (version 4.2.2; The R Foundation) and the threshold modeling was performed using the R *chngpt* package [43].

### Ethical Considerations

This study does not include human subjects research (no human subjects experimentation or intervention was conducted) and so does not require institutional review board approval.

## Results

The 2500 sets ranged from 1 to 200 disclosure statements with an average of 100 (SD 57.42). The number of sentences in each fine-tuning set ranged from 5 to 1031, with an average of 525.2 (SD 294.13). The tagged entity density ranged from 0.771 to 1.72 EPS, with an average of 1.34 (SD 0.14). Fine-tuned model performance on NER tasks ranged from *F*_1_-score=0.3 to *F*_1_-score=0.96. The top *F*_1_-score for each architecture was 0.72 for GPT-2_large, 0.92 for GatorTron_base, 0.94 for RoBERTa_base, and 0.96 for RoBERTa_large. Data set and model descriptive statistics are available in Table 2.

Multiple linear regressions were used to assess and compare the relationship between the independent variables (number of sentences and EPS) and the overall model performances (measured by *F*_1_-score) for each architecture. EPS and number of sentences predictors correlate weakly (Pearson *r*=0.28, *P*<.001), and diagnostic tests for multicollinearity indicate that the variables do not violate the Klein rule of thumb and have a low variance inflation score (1.11) and high tolerance (0.9) [44]. All models were statistically significant with multiple *R*^2^ ranging from 0.6057 to 0.7896 (all *P*<.001). EPS and the number of sentences were significant predictors of *F*_1_-scores in all cases (*P*<.001), except for the GPT-2_large model, where EPS was not a significant predictor (*P*=.184). Standardized regression coefficients and full model results are available in Table 3.

This study focuses primarily on total sentences as our measure of data size. This is because the number of sentences can be identified prospectively (prior to annotation) and is comparable across data sets with different document lengths. However, it should be noted that other measures of sample size are similarly predictive of *F*_1_-scores. The total number of relevant entities per training data set correlates very closely with the number of sentences (Pearson *r*=0.998, *P*<.001). This high collinearity makes it inadvisable to fit regression models with both predictors. We did, however, fit a series of models with EPS and a number of relevant entities as predictors. In all cases, the results were quite similar to those reported in Table 3. Specific values are available in Multimedia Appendix 2. It is notable that, in all cases, the multiple *R*^2^ for models with EPS and the number of relevant entities as predictors are lower than the counterpart models with EPS and number of sentences. Subsequent pairwise ANOVA, however, indicates that there are no significant differences in model fit. ANOVA *P* values were 0.85 for RoBERTa_base, 0.74 for GatorTron_base, 0.93 for RoBERTa_large, and 0.53 for GPT-2_large.

**Figure 1:**
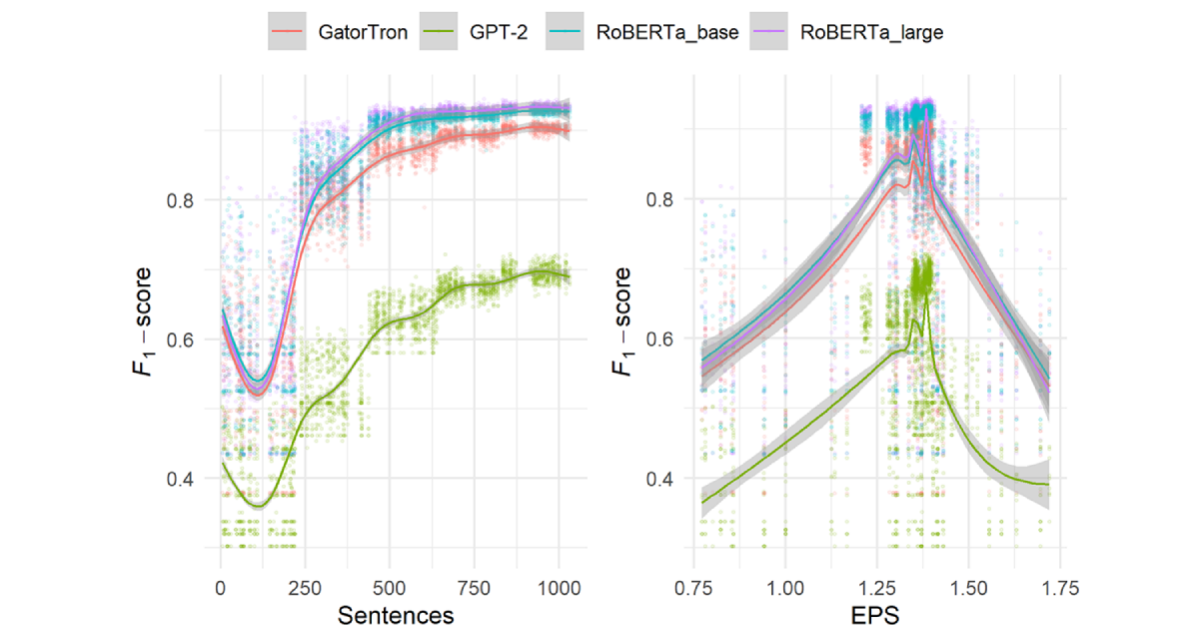
Single predictor plots for the number of sentence (left) and EPS (right). Fit with a generalized additive model. EPS: entities per sentence.

Threshold regression models were also used to assess the possibility of diminishing marginal returns on training data sizes and EPS for each model and model architecture. All threshold models indicate that there was a diminishing marginal return from increased training data set sample size measured by number of sentences. Point estimates ranged from 439 for RoBERTa_large to 527 for GPT-2_large. Likewise, the threshold models indicate a diminishing marginal return for EPS with point estimates between 1.36 and 1.38. Complete threshold regression results are available in Table 4. Single predictor plots are available in Figure 1, with technical threshold model plots shown in Multimedia Appendix 2.

**Table 2:** Descriptive statistics of training sets and model performance.

Descriptive statistics	Value, range	Value, mean (SD)
Number of disclosure statements	1-200	100.0 (57.42)
Number of tokens	4-1402	712.9 (405.94)
Number of sentences	5-1031	525.2 (294.13)
Entities per sentence	0.771-1.72	1.34 (0.14)
RoBERTa_base *F*_1_-score	0.43-0.94	0.81(0.13)
GatorTron_base *F*_1_-score	0.37-0.92	0.84 (0.13)
RoBERTa_large *F*_1_-score	0.44-0.96	0.84 (0.14)
GPT-2_large *F*_1_-score	0.30-0.72	0.58 (0.12)

**Table 3:** Standardized multiple linear regression results by architecture.

Model (parameters)	β_EPS_^a^	β_sent_	*F* test (*df*)	*P* value^b^	Multiple *R*^2^
RoBERTa_base (125M)	0.04^c^	0.78^c^	2034 (22, 497)	<.001	0.6197
GatorTron_base (345M)	0.05^c^	0.79^c^	2236 (22, 497)	<.001	0.6417
RoBERTa_large (355M)	0.05^c^	0.76^c^	1918 (22, 497)	<.001	0.6057
GPT-2_large (774M)	–0.01	0.89^c^	4685 (22, 497)	<.001	0.7896

^a^EPS: entities per sentence.

^b^Individual predictor *P* values for Beta_sent were <.001 for all models. *P* values for Beta_EPS were <.001 in all cases except for the GPT-2_large model where EPS was not a significant predictor (*P*=.184)

^c^Predictor results are significant at the *P*<.01 level.

**Table 4:** Threshold regression point estimates and 95% confidence intervals for number of sentences and EPS^a^ by architecture.

Model (parameters)	Number of sent threshold, estimate (95% CI)	EPS threshold, estimate (95% CI)
RoBERTa_base (125M)	448 (437-456)	1.36 (1.35-1.37)
GatorTron_base (345M)	448 (409-456)	1.36 (1.36-1.38)
RoBERTa_large (355M)	439 (409-451)	1.36 (1.35-1.38)
GPT-2_large (774M)	527 (511-540)	1.38 (1.36-1.38)

^a^EPS: entities per sentence.

## Discussion

### Principal Findings

Our review of the available literature on human-annotated training data for NER fine-tuning indicates that there is a strong need for useful guidance on requisite sample sizes. Reported sample units and sizes vary widely, providing little foundation for prospective approaches to sample curation. Given the significant time and costs associated with gold-standard annotation, it is critical that researchers and practitioners can effectively determine appropriate samples before fine-tuning neural network language models. The results of the experiment presented here provide initial actionable guidance for the development of gold-standard annotated training sets for NER fine-tuning in highly specific, specialized domains. Specifically, they indicate that contrary to common assumptions, transformer-based language models can be optimized for new tasks using relatively small amounts of training data. Furthermore, the results presented here indicate that NER fine-tuning is subject to threshold effects whereby there are diminishing marginal returns from increased sample sizes. Our data revealed that a scant 439 sentences were sufficient to reach that threshold with RoBERTa_large. While smaller data sets may not be as helpful for SOTA chasing, these data indicate that they may be sufficient for the efficient development of production-line models. These findings are consistent with the growing multidisciplinary body of literature demonstrating the efficacy of smaller sample sizes for fine-tuning [13,23,24]. Additionally, we note that given prior estimates for NER annotation rates, a sample of approximately 450 sentences would take between 74 and 225 minutes to annotate [8].

Importantly, the data provided here also indicate that neither model size nor content area–specific foundational training data may be essential for maximizing performance, but that model architecture is. RoBERTa_base, GatorTron_base, and RoBERTa_large all achieved comparable performance levels in terms of maximum *F*_1_-score with similarly low training sample sizes. GPT-2_large, despite being the largest model tested, showed the worst performance on our NER tasks. On the one hand, neither finding is surprising. The foundational paper by Devlin et al [16] on the BERT transformer architecture suggests that BERT’s capacity for fine-tuning for NLP tasks, such as classification, is better compared with GPT-based models, and a recent Microsoft Research paper argues that general-language models, such as GPT-4, can perform as well or better on domain-specific language tasks—specifically as they relate to medicine—than models trained on language specific to that domain [45]. But where the latter study focused on a very LLM built with reinforcement learning from human feedback and designed to be responsive to prompting, we found that for smaller—and therefore more tunable—models, fine-tuning with domain-specific texts yields significant performance improvements. For domain-specific NER tasks, then, architecture differences may matter most: decoder-based unidirectional architectures may be better suited for sentence generation, while encoder- or decoder-based bidirectional architectures better capture sentence-level contexts that are essential to NER tasks.

The results presented here also indicate that there are similar threshold effects for token density. That is, selecting or synthetically creating specifically token-rich samples may not improve model performance. Unlike the sample size data that indicate a diminishing marginal return, the hinge model for token density shows a substantial decrease in overall performance after the EPS threshold is achieved. We note that these threshold point estimates and narrow 95% CIs converge on the average EPS (1.34) of the 2500 training sets, and this suggests that the relevant entity density of training data needs to approximate the relevant entity density of testing and production-line data.

This finding is especially relevant given the increasing interest in artificial training data generated by LLMs. While the insights presented here indicate that fine-tuning training data can be much smaller than generally anticipated, high-quality small training data sets still require adequate funding and time to pay, train, and deploy human annotators. In response, some research seeks to leverage LLMs as sources of training data for subsequent fine-tuning of smaller neural network models [46]. This is an intriguing line of research worthy of further scrutiny. However, it is notable that our findings about relevant token density suggest that artificially generated data must mirror real data in terms of token density. If the token density is too low or too high, we can expect to see reduced model performance when compared with naturally derived training data and high-quality expert annotation.

While these findings provide an important initial foundation for fine-tuning sample size considerations in NER applications, the specifically identified thresholds may not apply to markedly different NER use cases. This study focused on fine-tuning PERSON and ORG tags, entity types that are well-represented across the heterogeneous data sources that are used to train LLMs. Bioinformatics use cases that focus on entity types that are more unique to biomedical contexts (eg, symptoms, chemicals, diseases, genes, and proteins) or that require generating new entity categories may require larger training samples to optimize LLM performance. Additionally, this study focuses on semistructured natural language (disclosure statements). While we would expect similar guidelines to apply for NER in other semistructured biomedical contexts (eg, research papers, clinical notes, abstracts, and figure or image annotations), the threshold guidance here may not apply well to less formalized linguistic contexts.

### Conclusion

The emergence of LLMs offers significant potential for improving NLP applications in biomedical informatics, with research demonstrating the advantages of fine-tuned, domain-specific language models for health care applications [47] and environmental costs [22]. However, given the novelty of these solutions, there is a general dearth of actionable guidelines on how to efficiently fine-tune language models. In the context of NER applications, this study demonstrates that there is a general lack of consensus and actionable guidance on sample size selection concerns for fine-tuning LLMs. Training sets reporting units and sample size varied widely in the published literature, with samples ranging from 100 sentences to 35,938 sentences for training sets. Additionally, human-annotated training set sample sizes are seldom justified or explained. In the rare cases where sample size is discussed explicitly, justifications focus narrowly on simple size comparisons to previously published efforts in a similar domain. In this context, biomedical informatics researchers could benefit from actionable guidelines about sample size considerations for fine-tuning LLMs.

The data presented here provide sample size guidance for fine-tuning LLMs drawn from an experiment on 2500 gold-standard human annotated fine-tuning samples. Specifically, the data demonstrate the importance of both sample sizes as measured in the number of sentences and relevant token density for training data curation. Furthermore, the findings indicate that both sample size and token density can be subject to threshold limitations where increased sample size or token density do not confer additional performance benefits. In this study, sample sizes of greater than 439-527 sentences failed to produce meaningful accuracy improvements. This suggests that researchers interested in levering LLMs for NER applications can save considerable time, effort, and funding, which has been historically devoted to producing gold-standard annotations. The data presented here also indicate that the relevant token density of training samples should reliably approximate the relevant token density of real-world cases. This finding has important ramifications for the production of synthetic data which may or may not effectively approximate real-world cases. The findings presented here can directly inform future research in health policy informatics and may also be applicable to a wider range of health and biomedical informatics tasks.

## Appendix

Multimedia Appendix 1 Review of sample sizes and justifications.

Multimedia Appendix 2 Detailed statistical results and threshold model plots.

## Abbreviations

BERT: Bidirectional Encoder Representations from Transformers
COI: conflicts of interest
EPS: entities per sentence
GPT: Generative Pre-trained Transformer
LLM: large language model
NER: named entity recognition
NLP: natural language processing
SOTA: state-of-the-art
USMLE: United States Medical Licensing Exam
