# Supplementary material for: Sample Size Considerations for Fine-Tuning Large Language Models for Named Entity Recognition Tasks: Methodological Study
Source: JMIR AI. 2024 May 16;3:e52095. doi: 10.2196/52095 (PMC11140272; doi:10.2196/52095)
Supplement: Multimedia Appendix 1 [file ai_v3i1e52095_app1.docx]

**Appendix 1: Review of Sample Sizes and Justifications**

**Table S1:** Sample unit, size, and justification from collected articles.

| Lead Author | Title | Journal | Year | size | Category | Justification |
| --- | --- | --- | --- | --- | --- | --- |
| Korvigo [1] | Putting hands to rest: efficient deep CNN-RNN architecture for chemical named entity recognition with no hand-crafted rules | J Cheminform | 2018 | 3,500 | abstracts |  |
| Abdelmageed [2] | BiodivNERE: Gold standard corpora for named entity recognition and relation extraction in the biodiversity domain | Biodivers Data J | 2022 | 2560 | sentences |  |
| Ananiadou [3] | Named entity recognition for bacterial Type IV secretion systems | PLoS One | 2011 | 14,351 | sentences |  |
| Bijari [4] | Assisted neuroscience knowledge extraction via machine learning applied to neural reconstruction metadata on NeuroMorpho.Org | Brain Inform | 2022 | 39,876 | entities |  |
| Campos [5] | A modular framework for biomedical concept recognition | BMC Bioinformatics | 2013 | 2,1749 | sentences |  |
| Campos [6] | Harmonization of gene/protein annotations: towards a gold standard MEDLINE | Bioinformatics | 2012 | 6,566 | abstracts/articles |  |
| Campos[7] | A document processing pipeline for annotating chemical entities in scientific documents | J Cheminform | 2015 | 3,500 | abstracts/articles |  |
| Catelli [8] | Crosslingual named entity recognition for clinical de-identification applied to a COVID-19 Italian data set | Appl Soft Comput | 2020 | 521 | clinical notes/reports |  |
| Chen [9] | Named entity recognition from Chinese adverse drug event reports with lexical feature based BiLSTM-CRF and tri-training | J Biomed Inform | 2019 | 15,000 | other |  |
| Chen [10] | A benchmark for automatic medical consultation system: frameworks, tasks and datasets | Bioinformatics | 2023 | 20 | abstracts/articles |  |
| Chew [11] | Identifying Electronic Nicotine Delivery System Brands and Flavors on Instagram: Natural Language Processing Analysis | J Med Internet Res | 2022 | 79,401 | tokens |  |
| Chun [12] | Automatic recognition of topic-classified relations between prostate cancer and genes using MEDLINE abstracts | BMC Bioinformatics | 2006 | 3,939 | sentences | Larger than prior study |
| Deng [13] | Constructing High-Fidelity Phenotype Knowledge Graphs for Infectious Diseases With a Fine-Grained Semantic Information Model: Development and Usability Study | J Med Internet Res | 2021 | 116 | other |  |
| Dhrangadhariya [14] | Not so weak PICO: leveraging weak supervision for participants, interventions, and outcomes recognition for systematic review automation | JAMIA Open | 2023 | 4933 | abstracts/articles |  |
| Dieb [15] | Framework for automatic information extraction from research papers on nanocrystal devices | Beilstein J Nanotechnol | 2015 | 392 | sentences |  |
| Doan [16] | Recognizing Medication related Entities in Hospital Discharge Summaries using Support Vector Machine | Proc Int Conf Comput Ling | 2010 | 17 | clinical notes/reports |  |
| Doğan [17] | NCBI disease corpus: a resource for disease name recognition and concept normalization | J Biomed Inform | 2014 | 593 | other |  |
| Du [18] | Extracting psychiatric stressors for suicide from social media using deep learning | BMC Med Inform Decis Mak | 2018 | 2,100 | other |  |
| Du [19] | Extracting postmarketing adverse events from safety reports in the vaccine adverse event reporting system (VAERS) using deep learning | J Am Med Inform Assoc | 2021 | 603 | sentences |  |
| Funnell [20] | Characterization of patients with idiopathic normal pressure hydrocephalus using natural language processing within an electronic healthcare record system | J Neurosurg | 2022 | 3,296 | other |  |
| Furrer [21] | OGER++: hybrid multi-type entity recognition | J Cheminform | 2019 | 47 | abstracts/articles | Larger than previous study |
| Giorgi [22] | Transfer learning for biomedical named entity recognition with neural networks | Bioinformatics | 2018 | 360,938 | sentences |  |
| Gobeill [23] | Overview of the BioCreative VI text-mining services for Kinome Curation Track | Database (Oxford) | 2018 | 100 | entities |  |
| Grego [24] | Chemical Entity Recognition and Resolution to ChEBI | ISRN Bioinform | 2012 | 40 | clinical notes/reports |  |
| Guo [25] | Evaluating automated entity extraction with respect to drug and non-drug treatment strategies | J Biomed Inform | 2019 | 1600 | abstracts/articles |  |
| Han [26] | Active learning for ontological event extraction incorporating named entity recognition and unknown word handling | J Biomed Semantics | 2016 | 360 | abstracts/articles |  |
| Hassanpour [27] | Information extraction from multi-institutional radiology reports | Artif Intell Med | 2016 | 150 | clinical notes/reports |  |
| Hu [28] | A Unified Model Using Distantly Supervised Data and Cross-Domain Data in NER | Comput Intell Neurosci | 2022 | 7,200 | sentences |  |
| Jiang [29] | A study of machine-learning-based approaches to extract clinical entities and their assertions from discharge summaries | J Am Med Inform Assoc | 2011 | 349 | clinical notes/reports |  |
| Johnson [30] | Deidentification of free-text medical records using pre-trained bidirectional transformers | Proc ACM Conf Health Inference Learn (2020) | 2020 | 1,304 | clinical notes/reports |  |
| Jonnalagadda [31] | Using empirically constructed lexical resources for named entity recognition | Biomed Inform Insights | 2013 | 15,000 | sentences |  |
| Kang [32] | EliIE: An open-source information extraction system for clinical trial eligibility criteria | J Am Med Inform Assoc | 2017 | 230 | abstracts/articles |  |
| Kaplar [33] | Evaluation of clinical named entity recognition methods for Serbian electronic health records | Int J Med Inform | 2022 | 6893 | sentences |  |
| Khare [34] | Automatic extraction of drug indications from FDA drug labels | AMIA Annu Symp Proc | 2014 | 500 | entities |  |
| Kim [35] | BioCreative V BioC track overview: collaborative biocurator assistant task for BioGRID | Database (Oxford) | 2016 | 110 | abstracts/articles |  |
| Klinger [36] | Detection of IUPAC and IUPAC-like chemical names | Bioinformatics | 2008 | 1,500 | sentences |  |
| Kongburan [37] | Enhancing metabolic event extraction performance with multitask learning concept | J Biomed Inform | 2019 | 143 | abstracts/articles |  |
| Korkontzelos [38] | Boosting drug named entity recognition using an aggregate classifier | Artif Intell Med | 2015 | 250 | abstracts/articles |  |
| Kormilitzin [39] | Med7: A transferable clinical natural language processing model for electronic health records | Artif Intell Med | 2021 | 1,212 | clinical notes/reports |  |
| Krallinger [40] | The CHEMDNER corpus of chemicals and drugs and its annotation principles | J Cheminform | 2015 | 3,500 | abstracts/articles |  |
| Krallinger [41] | CHEMDNER: The drugs and chemical names extraction challenge | J Cheminform | 2015 | 3,500 | abstracts/articles |  |
| Kühnel [42] | We are not ready yet: limitations of state-of-the-art disease named entity recognizers | J Biomed Semantics | 2022 | 649 | abstracts/articles |  |
| Leaman [43] | Challenges in clinical natural language processing for automated disorder normalization | J Biomed Inform | 2015 | 199 | clinical notes/reports |  |
| Lee [44] | Leveraging existing corpora for de-identification of psychiatric notes using domain adaptation | AMIA Annu Symp Proc | 2018 | 325 | clinical notes/reports |  |
| Lei [45] | A comprehensive study of named entity recognition in Chinese clinical text | J Am Med Inform Assoc | 2014 | 266 | clinical notes/reports |  |
| Lerner [46] | Terminologies augmented recurrent neural network model for clinical named entity recognition | J Biomed Inform | 2020 | 188 | clinical notes/reports |  |
| Lewinski [47] | An annotated corpus with nanomedicine and pharmacokinetic parameters | Int J Nanomedicine | 2017 | 28276 | sentences |  |
| Li [48] | Drug knowledge discovery via multi-task learning and pre-trained models | BMC Med Inform Decis Mak | 2021 | 2,027 | sentences |  |
| Liu [49] | De-identifying Australian hospital discharge summaries: An end-to-end framework using ensemble of deep learning models | J Biomed Inform | 2022 | 400 | clinical notes/reports |  |
| Liu. [50] | Automatic knowledge extraction from Chinese electronic medical records and rheumatoid arthritis knowledge graph construction | Quant Imaging Med Surg | 2023 | 367 | clinical notes/reports |  |
| Lossio-Ventura [51] | OC-2-KB: integrating crowdsourcing into an obesity and cancer knowledge base curation system | BMC Med Inform Decis Mak | 2018 | 671 | sentences |  |
| Macri [52] | Automated Identification of Clinical Procedures in Free-Text Electronic Clinical Records with a Low-Code Named Entity Recognition Workflow | Methods Inf Med | 2022 |  |  |  |
| Magge [53] | DeepADEMiner: a deep learning pharmacovigilance pipeline for extraction and normalization of adverse drug event mentions on Twitter | J Am Med Inform Assoc | 2021 | 25,678 | other |  |
| Miao [54] | Extraction of BI-RADS findings from breast ultrasound reports in Chinese using deep learning approaches | Int J Med Inform | 2018 | 360 | clinical notes/reports |  |
| Mithun [55] | Clinical Concept-Based Radiology Reports Classification Pipeline for Lung Carcinoma | J Digit Imaging | 2023 | 200 | clinical notes/reports |  |
| Moezzi [56] | Application of Deep Learning in Generating Structured Radiology Reports: A Transformer-Based Technique | J Digit Imaging | 2022 | 71 | clinical notes/reports |  |
| Nath [57] | The quest for better clinical word vectors: Ontology based and lexical vector augmentation versus clinical contextual embeddings | Comput Biol Med | 2021 | 47 | other |  |
| Paul [58] | Investigation of the Utility of Features in a Clinical De-identification Model: A Demonstration Using EHR Pathology Reports for Advanced NSCLC Patients | Front Digit Health | 2022 | 1,000 | clinical notes/reports |  |
| Peng [59] | Improving chemical disease relation extraction with rich features and weakly labeled data | J Cheminform | 2016 | 500 | abstracts/articles |  |
| Popovski [60] | FoodBase corpus: a new resource of annotated food entities | Database (Oxford) | 2019 | 1,000 | other |  |
| Pradhan [61] | Evaluating the state of the art in disorder recognition and normalization of the clinical narrative | J Am Med Inform Assoc | 2015 | 199 | clinical notes/reports |  |
| Richter-Pechanski [62] | Deep Learning Approaches Outperform Conventional Strategies in De-Identification of German Medical Reports | Stud Health Technol Inform | 2019 | 113 | clinical notes/reports |  |
| Rybinski [63] | Extracting Family History Information From Electronic Health Records: Natural Language Processing Analysis | JMIR Med Inform | 2021 | 117 | clinical notes/reports |  |
| Shardlow [64] | A Text Mining Pipeline Using Active and Deep Learning Aimed at Curating Information in Computational Neuroscience | Neuroinformatics | 2019 | 500 | sentences |  |
| Singh [65] | One clinician is all you need-cardiac magnetic resonance imaging measurement extraction: deep learning algorithm development | JMIR Med Inform | 2022 | 370 | clinical notes/reports |  |
| Skeppstedt [66] | Automatic recognition of disorders, findings, pharmaceuticals and body structures from clinical text: an annotation and machine learning study | J Biomed Inform | 2014 | 45,482 | tokens |  |
| Soysal [67] | CLAMP - a toolkit for efficiently building customized clinical natural language processing pipelines | J Am Med Inform Assoc | 2018 | 100 | sentences |  |
| Steinkamp [68] | Task definition, annotated dataset, and supervised natural language processing models for symptom extraction from unstructured clinical notes | J Biomed Inform | 2020 | 1,008 | clinical notes/reports |  |
| Steinkamp [69] | Evaluation of Automated Public De-Identification Tools on a Corpus of Radiology Reports) | Radiol Artif Intell | 2020 | 1,480 | tokens |  |
| Stylianou [70] | EBM+: Advancing Evidence-Based Medicine via two level automatic identification of Populations, Interventions, Outcomes in medical literature | Artif Intell Med | 2020 | 130 | abstracts/articles |  |
| Sugimoto [71] | Extracting clinical information from japanese radiology reports using a 2-stage deep learning approach: algorithm development and validation | JMIR Med Inform | 2023 | 1040 | clinical notes/reports |  |
| Sun [72] | Leveraging a joint learning model to extract mixture symptom mentions from traditional Chinese medicine clinical notes | Biomed Res Int | 2022 | 2255 | clinical notes/reports |  |
| Sun J [73] | Deep learning-based methods for natural hazard named entity recognition | Sci Rep | 2022 | 806 | sentences |  |
| Tan [74] | Combining machine learning with a rule-based algorithm to detect and identify related entities of documented adverse drug reactions on hospital discharge summaries | Drug Saf | 2022 | 4020 | clinical notes/reports |  |
| Tang [75] | A comparison of conditional random fields and structured support vector machines for chemical entity recognition in biomedical literature | J Cheminform | 2015 | 3,500 | abstracts/articles |  |
| Tejani [76] | Performance of Multiple Pretrained BERT Models to Automate and Accelerate Data Annotation for Large Datasets | Radiol Artif Intell | 2022 | 1,004 | clinical notes/reports |  |
| Thieu [77] | A comprehensive study of mobility functioning information in clinical notes: Entity hierarchy, corpus annotation, and sequence labeling | Int J Med Inform | 2021 | 400 | clinical notes/reports |  |
| Tsuruoka [78] | Accelerating the annotation of sparse named entities by dynamic sentence selection | BMC Bioinformatics | 2008 | 18,546 | sentences |  |
| Wagholikar [79] | Pooling annotated corpora for clinical concept extraction | J Biomed Semantics | 2013 | 349 | clinical notes/reports |  |
| Wang [80] | PICO entity extraction for preclinical animal literature | Syst Rev | 2022 | 3,398 | sentences |  |
| Weegar [81] | Finding Cervical Cancer Symptoms in Swedish Clinical Text using a Machine Learning Approach and NegEx | AMIA Annu Symp Proc | 2015 | 180 | clinical notes/reports |  |
| Wei [82] | A study of deep learning approaches for medication and adverse drug event extraction from clinical text | J Am Med Inform Assoc | 2020 | 303 | clinical notes/reports |  |
| Wei [83] | Recognizing software names in biomedical literature using machine learning | Health Informatics J | 2020 | 746 | abstracts/articles |  |
| Wei [84] | Assessing the state of the art in biomedical relation extraction: overview of the BioCreative V chemical-disease relation (CDR) task | Database (Oxford) | 2016 | 500 | abstracts/articles |  |
| Yang [85] | Transformers-sklearn: a toolkit for medical language understanding with transformer-based models | BMC Med Inform Decis Mak | 2021 | 7,645 | entities |  |
| Yang [86] | Identifying relations of medications with adverse drug events using recurrent convolutional neural networks and gradient boosting | J Am Med Inform Assoc | 2020 | 303 | clinical notes/reports |  |
| Yim [87] | Tumor information extraction in radiology reports for hepatocellular carcinoma patients | AMIA Jt Summits Transl Sci Proc | 2016 | 101 | clinical notes/reports |  |
| Zaman [88] | Automatic Diagnosis Labeling of Cardiovascular MRI by Using Semisupervised Natural Language Processing of Text Reports | Radiol Artif Intell | 2021 | 801 | clinical notes/reports |  |
| Zhang [89] | Clinical Named Entity Recognition From Chinese Electronic Health Records via Machine Learning Methods | JMIR Med Inform | 2018 | 29,866 | entities |  |
| Zhang E [90] | Improving Clinical Named-Entity Recognition with Transfer Learning | Stud Health Technol Inform | 2018 | 199 | clinical notes/reports |  |
| Zhang L [91] | Lexicon and attention-based named entity recognition for kiwifruit diseases and pests: A Deep learning approach | Front Plant Sci | 2022 | 61,103 | sentences |  |
| Zhang Y [92] | Chemical named entity recognition in patents by domain knowledge and unsupervised feature learning | Database (Oxford) | 2016 | 7000 | abstracts/articles | Larger than previous study |
| Zhou [93] | CancerBERT: a cancer domain-specific language model for extracting breast cancer phenotypes from electronic health records | J Am Med Inform Assoc | 2022 | 9,685 | sentences |  |

**Supplementary References**

1. Korvigo I, Holmatov M, Zaikovskii A, Skoblov M. Putting hands to rest: efficient deep CNN-RNN architecture for chemical named entity recognition with no hand-crafted rules. J Cheminformatics. 2018 May 23;10(1):28.

2. Abdelmageed N, Löffler F, Feddoul L, Algergawy A, Samuel S, Gaikwad J, et al. BiodivNERE: Gold standard corpora for named entity recognition and relation extraction in the biodiversity domain. Biodivers Data J. 2022;10:e89481.

3. Ananiadou S, Sullivan D, Black W, Levow GA, Gillespie JJ, Mao C, et al. Named entity recognition for bacterial Type IV secretion systems. PloS One. 2011 Mar 29;6(3):e14780.

4. Bijari K, Zoubi Y, Ascoli GA. Assisted neuroscience knowledge extraction via machine learning applied to neural reconstruction metadata on NeuroMorpho.Org. Brain Inform. 2022 Nov 7;9(1):26.

5. Campos D, Matos S, Oliveira JL. A modular framework for biomedical concept recognition. BMC Bioinformatics. 2013 Sep 24;14:281.

6. Campos D, Matos S, Lewin I, Oliveira JL, Rebholz-Schuhmann D. Harmonization of gene/protein annotations: towards a gold standard MEDLINE. Bioinforma Oxf Engl. 2012 May 1;28(9):1253–61.

7. Campos D, Matos S, Oliveira JL. A document processing pipeline for annotating chemical entities in scientific documents. J Cheminformatics. 2015;7(Suppl 1 Text mining for chemistry and the CHEMDNER track):S7.

8. Catelli R, Gargiulo F, Casola V, De Pietro G, Fujita H, Esposito M. Crosslingual named entity recognition for clinical de-identification applied to a COVID-19 Italian data set. Appl Soft Comput. 2020 Dec;97:106779.

9. Chen Y, Zhou C, Li T, Wu H, Zhao X, Ye K, et al. Named entity recognition from Chinese adverse drug event reports with lexical feature based BiLSTM-CRF and tri-training. J Biomed Inform. 2019 Aug;96:103252.

10. Chen W, Li Z, Fang H, Yao Q, Zhong C, Hao J, et al. A benchmark for automatic medical consultation system: frameworks, tasks and datasets. Bioinforma Oxf Engl. 2023 Jan 1;39(1):btac817.

11. Chew R, Wenger M, Guillory J, Nonnemaker J, Kim A. Identifying Electronic Nicotine Delivery System Brands and Flavors on Instagram: Natural Language Processing Analysis. J Med Internet Res. 2022 Jan 18;24(1):e30257.

12. Chun HW, Tsuruoka Y, Kim JD, Shiba R, Nagata N, Hishiki T, et al. Automatic recognition of topic-classified relations between prostate cancer and genes using MEDLINE abstracts. BMC Bioinformatics. 2006 Nov 24;7 Suppl 3(Suppl 3):S4.

13. Deng L, Chen L, Yang T, Liu M, Li S, Jiang T. Constructing High-Fidelity Phenotype Knowledge Graphs for Infectious Diseases With a Fine-Grained Semantic Information Model: Development and Usability Study. J Med Internet Res. 2021 Jun 15;23(6):e26892.

14. Dhrangadhariya A, Müller H. Not so weak PICO: leveraging weak supervision for participants, interventions, and outcomes recognition for systematic review automation. JAMIA Open. 2023 Apr;6(1):ooac107.

15. Dieb TM, Yoshioka M, Hara S, Newton MC. Framework for automatic information extraction from research papers on nanocrystal devices. Beilstein J Nanotechnol. 2015;6:1872–82.

16. Doan S, Xu H. Recognizing Medication related Entities in Hospital Discharge Summaries using Support Vector Machine. Proc COLING Int Conf Comput Linguist. 2010 Aug;2010:259–66.

17. Doğan RI, Leaman R, Lu Z. NCBI disease corpus: a resource for disease name recognition and concept normalization. J Biomed Inform. 2014 Feb;47:1–10.

18. Du J, Zhang Y, Luo J, Jia Y, Wei Q, Tao C, et al. Extracting psychiatric stressors for suicide from social media using deep learning. BMC Med Inform Decis Mak. 2018 Jul 23;18(Suppl 2):43.

19. Du J, Xiang Y, Sankaranarayanapillai M, Zhang M, Wang J, Si Y, et al. Extracting postmarketing adverse events from safety reports in the vaccine adverse event reporting system (VAERS) using deep learning. J Am Med Inform Assoc JAMIA. 2021 Jul 14;28(7):1393–400.

20. Funnell JP, Noor K, Khan DZ, D’Antona L, Dobson RJB, Hanrahan JG, et al. Characterization of patients with idiopathic normal pressure hydrocephalus using natural language processing within an electronic healthcare record system. J Neurosurg. 2022 Nov 18;1–9.

21. Furrer L, Jancso A, Colic N, Rinaldi F. OGER++: hybrid multi-type entity recognition. J Cheminformatics. 2019 Jan 21;11(1):7.

22. Giorgi JM, Bader GD. Transfer learning for biomedical named entity recognition with neural networks. Bioinforma Oxf Engl. 2018 Dec 1;34(23):4087–94.

23. Gobeill J, Gaudet P, Dopp D, Morrone A, Kahanda I, Hsu YY, et al. Overview of the BioCreative VI text-mining services for Kinome Curation Track. Database J Biol Databases Curation. 2018 Jan 1;2018:bay104.

24. Grego T, Pesquita C, Bastos HP, Couto FM. Chemical Entity Recognition and Resolution to ChEBI. ISRN Bioinforma. 2012;2012:619427.

25. Guo J, Blake C, Guan Y. Evaluating automated entity extraction with respect to drug and non-drug treatment strategies. J Biomed Inform. 2019 Jun;94:103177.

26. Han X, Kim J jae, Kwoh CK. Active learning for ontological event extraction incorporating named entity recognition and unknown word handling. J Biomed Semant. 2016;7:22.

27. Hassanpour S, Langlotz CP. Information extraction from multi-institutional radiology reports. Artif Intell Med. 2016 Jan;66:29–39.

28. Hu Y, He H, Chen Z, Zhu Q, Zheng C. A Unified Model Using Distantly Supervised Data and Cross-Domain Data in NER. Comput Intell Neurosci. 2022;2022:1987829.

29. Jiang M, Chen Y, Liu M, Rosenbloom ST, Mani S, Denny JC, et al. A study of machine-learning-based approaches to extract clinical entities and their assertions from discharge summaries. J Am Med Inform Assoc JAMIA. 2011;18(5):601–6.

30. Johnson AEW, Bulgarelli L, Pollard TJ. Deidentification of free-text medical records using pre-trained bidirectional transformers. Proc ACM Conf Health Inference Learn. 2020 Apr;2020:214–21.

31. Jonnalagadda S, Cohen T, Wu S, Liu H, Gonzalez G. Using empirically constructed lexical resources for named entity recognition. Biomed Inform Insights. 2013;6(Suppl 1):17–27.

32. Kang T, Zhang S, Tang Y, Hruby GW, Rusanov A, Elhadad N, et al. EliIE: An open-source information extraction system for clinical trial eligibility criteria. J Am Med Inform Assoc JAMIA. 2017 Nov 1;24(6):1062–71.

33. Kaplar A, Stošović M, Kaplar A, Brković V, Naumović R, Kovačević A. Evaluation of clinical named entity recognition methods for Serbian electronic health records. Int J Med Inf. 2022 Aug;164:104805.

34. Khare R, Wei CH, Lu Z. Automatic extraction of drug indications from FDA drug labels. AMIA Annu Symp Proc AMIA Symp. 2014;2014:787–94.

35. Kim S, Islamaj Doğan R, Chatr-Aryamontri A, Chang CS, Oughtred R, Rust J, et al. BioCreative V BioC track overview: collaborative biocurator assistant task for BioGRID. Database J Biol Databases Curation. 2016;2016:baw121.

36. Klinger R, Kolárik C, Fluck J, Hofmann-Apitius M, Friedrich CM. Detection of IUPAC and IUPAC-like chemical names. Bioinforma Oxf Engl. 2008 Jul 1;24(13):i268-276.

37. Kongburan W, Padungweang P, Krathu W, Chan JH. Enhancing metabolic event extraction performance with multitask learning concept. J Biomed Inform. 2019 May;93:103156.

38. Korkontzelos I, Piliouras D, Dowsey AW, Ananiadou S. Boosting drug named entity recognition using an aggregate classifier. Artif Intell Med. 2015 Oct;65(2):145–53.

39. Kormilitzin A, Vaci N, Liu Q, Nevado-Holgado A. Med7: A transferable clinical natural language processing model for electronic health records. Artif Intell Med. 2021 Aug;118:102086.

40. Krallinger M, Rabal O, Leitner F, Vazquez M, Salgado D, Lu Z, et al. The CHEMDNER corpus of chemicals and drugs and its annotation principles. J Cheminformatics. 2015;7(Suppl 1 Text mining for chemistry and the CHEMDNER track):S2.

41. Krallinger M, Leitner F, Rabal O, Vazquez M, Oyarzabal J, Valencia A. CHEMDNER: The drugs and chemical names extraction challenge. J Cheminformatics. 2015;7(Suppl 1 Text mining for chemistry and the CHEMDNER track):S1.

42. Kühnel L, Fluck J. We are not ready yet: limitations of state-of-the-art disease named entity recognizers. J Biomed Semant. 2022 Oct 27;13(1):26.

43. Leaman R, Khare R, Lu Z. Challenges in clinical natural language processing for automated disorder normalization. J Biomed Inform. 2015 Oct;57:28–37.

44. Lee HJ, Zhang Y, Roberts K, Xu H. Leveraging existing corpora for de-identification of psychiatric notes using domain adaptation. AMIA Annu Symp Proc AMIA Symp. 2017;2017:1070–9.

45. Lei J, Tang B, Lu X, Gao K, Jiang M, Xu H. A comprehensive study of named entity recognition in Chinese clinical text. J Am Med Inform Assoc JAMIA. 2014;21(5):808–14.

46. Lerner I, Paris N, Tannier X. Terminologies augmented recurrent neural network model for clinical named entity recognition. J Biomed Inform. 2020 Feb;102:103356.

47. Lewinski NA, Jimenez I, McInnes BT. An annotated corpus with nanomedicine and pharmacokinetic parameters. Int J Nanomedicine. 2017;12:7519–27.

48. Li D, Xiong Y, Hu B, Tang B, Peng W, Chen Q. Drug knowledge discovery via multi-task learning and pre-trained models. BMC Med Inform Decis Mak. 2021 Nov 16;21(Suppl 9):251.

49. Liu L, Perez-Concha O, Nguyen A, Bennett V, Jorm L. De-identifying Australian hospital discharge summaries: An end-to-end framework using ensemble of deep learning models. J Biomed Inform. 2022 Nov;135:104215.

50. Liu F, Liu M, Li M, Xin Y, Gao D, Wu J, et al. Automatic knowledge extraction from Chinese electronic medical records and rheumatoid arthritis knowledge graph construction. Quant Imaging Med Surg. 2023 Jun 1;13(6):3873–90.

51. Lossio-Ventura JA, Hogan W, Modave F, Guo Y, He Z, Yang X, et al. OC-2-KB: integrating crowdsourcing into an obesity and cancer knowledge base curation system. BMC Med Inform Decis Mak. 2018 Jul 23;18(Suppl 2):55.

52. Macri C, Teoh I, Bacchi S, Sun M, Selva D, Casson R, et al. Automated Identification of Clinical Procedures in Free-Text Electronic Clinical Records with a Low-Code Named Entity Recognition Workflow. Methods Inf Med. 2022 Sep;61(3–04):84–9.

53. Magge A, Tutubalina E, Miftahutdinov Z, Alimova I, Dirkson A, Verberne S, et al. DeepADEMiner: a deep learning pharmacovigilance pipeline for extraction and normalization of adverse drug event mentions on Twitter. J Am Med Inform Assoc JAMIA. 2021 Sep 18;28(10):2184–92.

54. Miao S, Xu T, Wu Y, Xie H, Wang J, Jing S, et al. Extraction of BI-RADS findings from breast ultrasound reports in Chinese using deep learning approaches. Int J Med Inf. 2018 Nov;119:17–21.

55. Mithun S, Jha AK, Sherkhane UB, Jaiswar V, Purandare NC, Dekker A, et al. Clinical Concept-Based Radiology Reports Classification Pipeline for Lung Carcinoma. J Digit Imaging. 2023 Feb 14;

56. Moezzi SAR, Ghaedi A, Rahmanian M, Mousavi SZ, Sami A. Application of Deep Learning in Generating Structured Radiology Reports: A Transformer-Based Technique. J Digit Imaging. 2023 Feb;36(1):80–90.

57. Nath N, Lee SH, McDonnell MD, Lee I. The quest for better clinical word vectors: Ontology based and lexical vector augmentation versus clinical contextual embeddings. Comput Biol Med. 2021 Jul;134:104433.

58. Paul T, Rana MKZ, Tautam PA, Kotapati TVP, Jampani Y, Singh N, et al. Investigation of the Utility of Features in a Clinical De-identification Model: A Demonstration Using EHR Pathology Reports for Advanced NSCLC Patients. Front Digit Health. 2022;4:728922.

59. Peng Y, Wei CH, Lu Z. Improving chemical disease relation extraction with rich features and weakly labeled data. J Cheminformatics. 2016;8:53.

60. Popovski G, Seljak BK, Eftimov T. FoodBase corpus: a new resource of annotated food entities. Database J Biol Databases Curation. 2019 Jan 1;2019:baz121.

61. Pradhan S, Elhadad N, South BR, Martinez D, Christensen L, Vogel A, et al. Evaluating the state of the art in disorder recognition and normalization of the clinical narrative. J Am Med Inform Assoc JAMIA. 2015 Jan;22(1):143–54.

62. Richter-Pechanski P, Amr A, Katus HA, Dieterich C. Deep Learning Approaches Outperform Conventional Strategies in De-Identification of German Medical Reports. Stud Health Technol Inform. 2019 Sep 3;267:101–9.

63. Rybinski M, Dai X, Singh S, Karimi S, Nguyen A. Extracting Family History Information From Electronic Health Records: Natural Language Processing Analysis. JMIR Med Inform. 2021 Apr 30;9(4):e24020.

64. Shardlow M, Ju M, Li M, O’Reilly C, Iavarone E, McNaught J, et al. A Text Mining Pipeline Using Active and Deep Learning Aimed at Curating Information in Computational Neuroscience. Neuroinformatics. 2019 Jul;17(3):391–406.

65. Singh P, Haimovich J, Reeder C, Khurshid S, Lau ES, Cunningham JW, et al. One Clinician Is All You Need-Cardiac Magnetic Resonance Imaging Measurement Extraction: Deep Learning Algorithm Development. JMIR Med Inform. 2022 Sep 16;10(9):e38178.

66. Skeppstedt M, Kvist M, Nilsson GH, Dalianis H. Automatic recognition of disorders, findings, pharmaceuticals and body structures from clinical text: an annotation and machine learning study. J Biomed Inform. 2014 Jun;49:148–58.

67. Soysal E, Wang J, Jiang M, Wu Y, Pakhomov S, Liu H, et al. CLAMP - a toolkit for efficiently building customized clinical natural language processing pipelines. J Am Med Inform Assoc JAMIA. 2018 Mar 1;25(3):331–6.

68. Steinkamp JM, Bala W, Sharma A, Kantrowitz JJ. Task definition, annotated dataset, and supervised natural language processing models for symptom extraction from unstructured clinical notes. J Biomed Inform. 2020 Feb;102:103354.

69. Steinkamp JM, Pomeranz T, Adleberg J, Kahn CE, Cook TS. Evaluation of Automated Public De-Identification Tools on a Corpus of Radiology Reports. Radiol Artif Intell. 2020 Nov;2(6):e190137.

70. Stylianou N, Razis G, Goulis DG, Vlahavas I. EBM+: Advancing Evidence-Based Medicine via two level automatic identification of Populations, Interventions, Outcomes in medical literature. Artif Intell Med. 2020 Aug;108:101949.

71. Sugimoto K, Wada S, Konishi S, Okada K, Manabe S, Matsumura Y, et al. Extracting Clinical Information From Japanese Radiology Reports Using a 2-Stage Deep Learning Approach: Algorithm Development and Validation. JMIR Med Inform. 2023 Nov 14;11:e49041.

72. Sun Y, Zhao Z, Wang Z, He H, Guo F, Luo Y, et al. Leveraging a Joint learning Model to Extract Mixture Symptom Mentions from Traditional Chinese Medicine Clinical Notes. BioMed Res Int. 2022;2022:2146236.

73. Sun J, Liu Y, Cui J, He H. Deep learning-based methods for natural hazard named entity recognition. Sci Rep. 2022 Mar 17;12(1):4598.

74. Tan HX, Teo CHD, Ang PS, Loke WPC, Tham MY, Tan SH, et al. Combining Machine Learning with a Rule-Based Algorithm to Detect and Identify Related Entities of Documented Adverse Drug Reactions on Hospital Discharge Summaries. Drug Saf. 2022 Aug;45(8):853–62.

75. Tang B, Feng Y, Wang X, Wu Y, Zhang Y, Jiang M, et al. A comparison of conditional random fields and structured support vector machines for chemical entity recognition in biomedical literature. J Cheminformatics. 2015;7(Suppl 1 Text mining for chemistry and the CHEMDNER track):S8.

76. Tejani AS, Ng YS, Xi Y, Fielding JR, Browning TG, Rayan JC. Performance of Multiple Pretrained BERT Models to Automate and Accelerate Data Annotation for Large Datasets. Radiol Artif Intell. 2022 Jul;4(4):e220007.

77. Thieu T, Maldonado JC, Ho PS, Ding M, Marr A, Brandt D, et al. A comprehensive study of mobility functioning information in clinical notes: Entity hierarchy, corpus annotation, and sequence labeling. Int J Med Inf. 2021 Mar;147:104351.

78. Tsuruoka Y, Tsujii J, Ananiadou S. Accelerating the annotation of sparse named entities by dynamic sentence selection. BMC Bioinformatics. 2008 Nov 19;9 Suppl 11(Suppl 11):S8.

79. Wagholikar KB, Torii M, Jonnalagadda SR, Liu H. Pooling annotated corpora for clinical concept extraction. J Biomed Semant. 2013 Jan 8;4(1):3.

80. Wang Q, Liao J, Lapata M, Macleod M. PICO entity extraction for preclinical animal literature. Syst Rev. 2022 Sep 30;11(1):209.

81. Weegar R, Kvist M, Sundström K, Brunak S, Dalianis H. Finding Cervical Cancer Symptoms in Swedish Clinical Text using a Machine Learning Approach and NegEx. AMIA Annu Symp Proc AMIA Symp. 2015;2015:1296–305.

82. Wei Q, Ji Z, Li Z, Du J, Wang J, Xu J, et al. A study of deep learning approaches for medication and adverse drug event extraction from clinical text. J Am Med Inform Assoc JAMIA. 2020 Jan 1;27(1):13–21.

83. Wei Q, Zhang Y, Amith M, Lin R, Lapeyrolerie J, Tao C, et al. Recognizing software names in biomedical literature using machine learning. Health Informatics J. 2020 Mar;26(1):21–33.

84. Wei CH, Peng Y, Leaman R, Davis AP, Mattingly CJ, Li J, et al. Assessing the state of the art in biomedical relation extraction: overview of the BioCreative V chemical-disease relation (CDR) task. Database J Biol Databases Curation. 2016;2016:baw032.

85. Yang F, Wang X, Ma H, Li J. Transformers-sklearn: a toolkit for medical language understanding with transformer-based models. BMC Med Inform Decis Mak. 2021 Jul 30;21(Suppl 2):90.

86. Yang X, Bian J, Fang R, Bjarnadottir RI, Hogan WR, Wu Y. Identifying relations of medications with adverse drug events using recurrent convolutional neural networks and gradient boosting. J Am Med Inform Assoc JAMIA. 2020 Jan 1;27(1):65–72.

87. Yim WW, Denman T, Kwan SW, Yetisgen M. Tumor information extraction in radiology reports for hepatocellular carcinoma patients. AMIA Jt Summits Transl Sci Proc AMIA Jt Summits Transl Sci. 2016;2016:455–64.

88. Zaman S, Petri C, Vimalesvaran K, Howard J, Bharath A, Francis D, et al. Automatic Diagnosis Labeling of Cardiovascular MRI by Using Semisupervised Natural Language Processing of Text Reports. Radiol Artif Intell. 2022 Jan;4(1):e210085.

89. Zhang Y, Wang X, Hou Z, Li J. Clinical Named Entity Recognition From Chinese Electronic Health Records via Machine Learning Methods. JMIR Med Inform. 2018 Dec 17;6(4):e50.

90. Zhang E, Thurier Q, Boyle L. Improving Clinical Named-Entity Recognition with Transfer Learning. Stud Health Technol Inform. 2018;252:182–7.

91. Zhang L, Nie X, Zhang M, Gu M, Geissen V, Ritsema CJ, et al. Lexicon and attention-based named entity recognition for kiwifruit diseases and pests: A Deep learning approach. Front Plant Sci. 2022;13:1053449.

92. Zhang Y, Xu J, Chen H, Wang J, Wu Y, Prakasam M, et al. Chemical named entity recognition in patents by domain knowledge and unsupervised feature learning. Database J Biol Databases Curation. 2016;2016:baw049.

93. Zhou S, Wang N, Wang L, Liu H, Zhang R. CancerBERT: a cancer domain-specific language model for extracting breast cancer phenotypes from electronic health records. J Am Med Inform Assoc JAMIA. 2022 Jun 14;29(7):1208–16.
