# Supplementary material for: Sample Size Considerations for Fine-Tuning Large Language Models for Named Entity Recognition Tasks: Methodological Study
Source: JMIR AI. 2024 May 16;3:e52095. doi: 10.2196/52095 (PMC11140272; doi:10.2196/52095)
Supplement: Multimedia Appendix 2 [file ai_v3i1e52095_app2.docx]

**Appendix 2: Detailed Statistical Results and Threshold Model Plots**

**Table S1: Comparison of Multiple Linear Regression Models By Predictor Variables and Architecture**

| Architecture (Params) | EPS + N Sentences | EPS + N Relevant Entities |
| --- | --- | --- |
| RoBERTa  (125M) | F(2,2497) = 2034, p <0.001, R^2^= 0.6197 | F(2,2496) = 1881, p < 0.01, R2 = 0.601 |
| GatorTron  (345M) | F(2,2497) = 2236, p <0.001, R^2^= 0.6417 | F(2,2496) = 2078, p < 0.01, R2 = 0.5247 |
| RoBERTa  (355M) | F(2,2497) = 1918, p <0.001, R^2^= 0.6057 | F(2,2496) = 1774, p < 0.01, R2 = 0.5869 |
| GPT-2  (774M) | F(2,2497) = 4685, p <0.001, R^2^= 0.7896 | F(2,2496) = 4288, p < 0.01, R2 = 0.7745 |

**
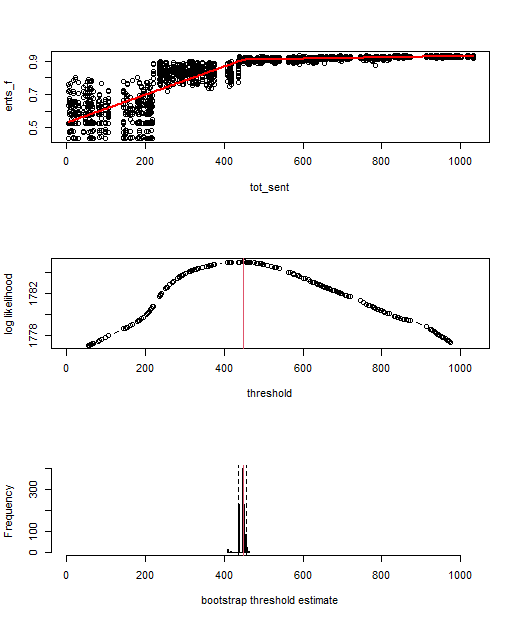
**

**Figure S1.** RoBERTa_base Threshold model for N of Sentences.

**
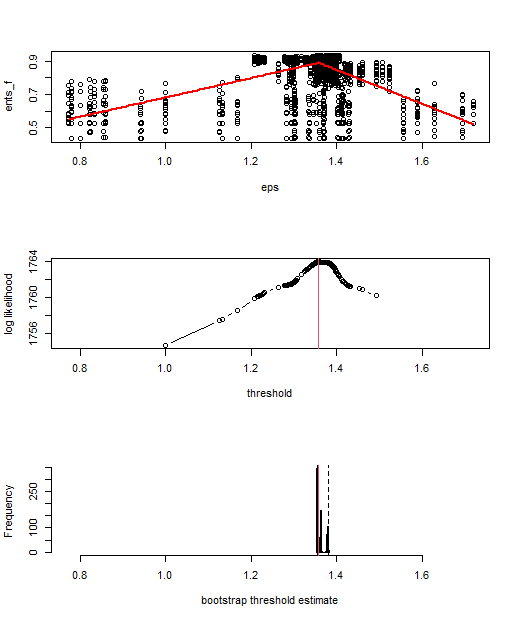
**

**Figure S2.** RoBERTa_base Threshold model for EPS

**
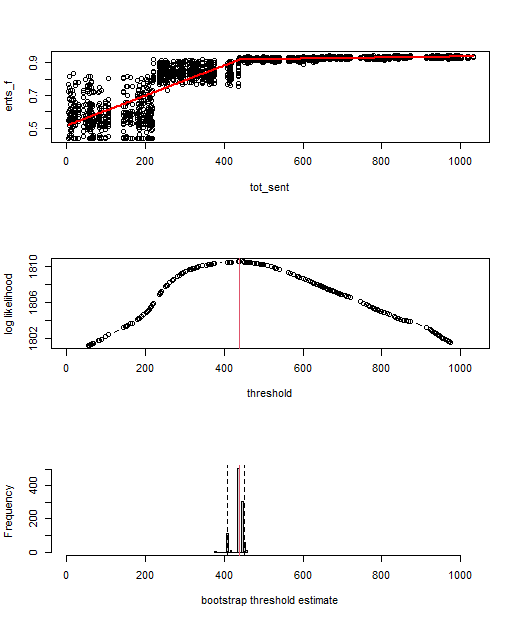
**

**Figure S3.** RoBERTa_large Threshold model for N of Sentences.


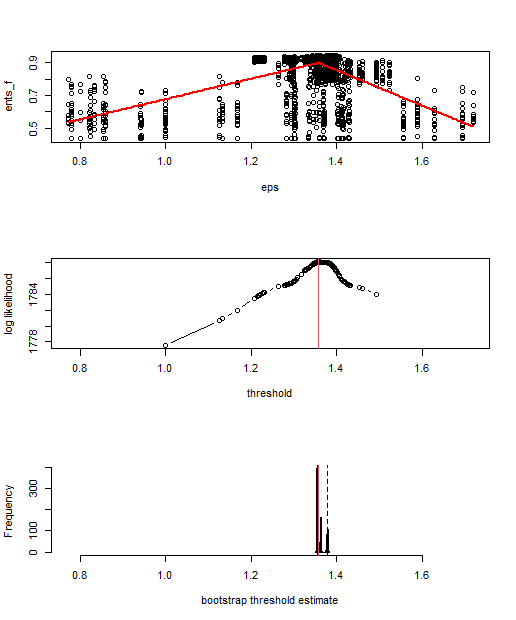


**Figure S4.** RoBERTa_large Threshold model for EPS


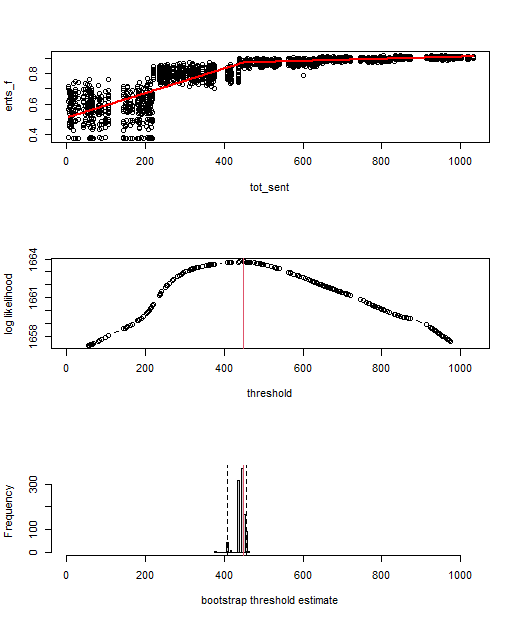


**Figure S5.** GatorTron Threshold model for N of Sentences.


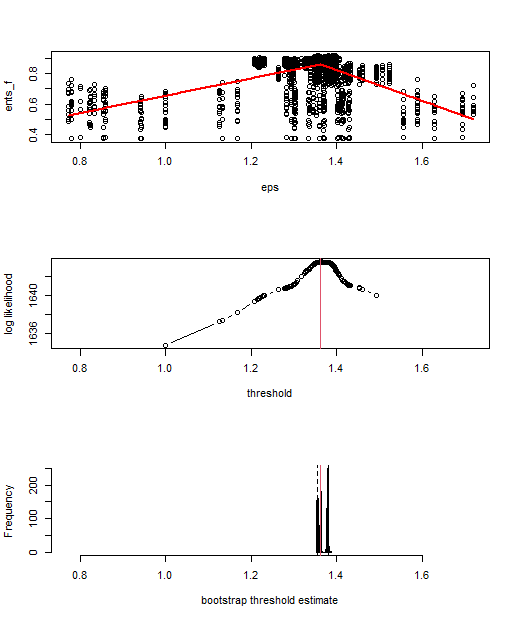


**Figure S6.** GatorTron Threshold model for EPS


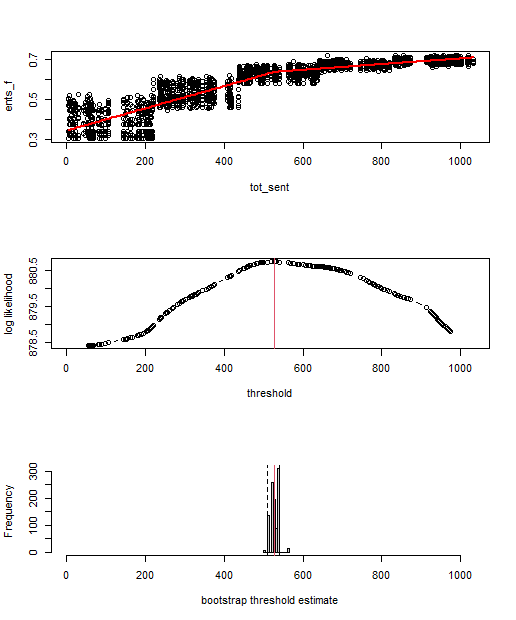


**Figure S7.** GPT-2 Threshold model for N of Sentences.

**
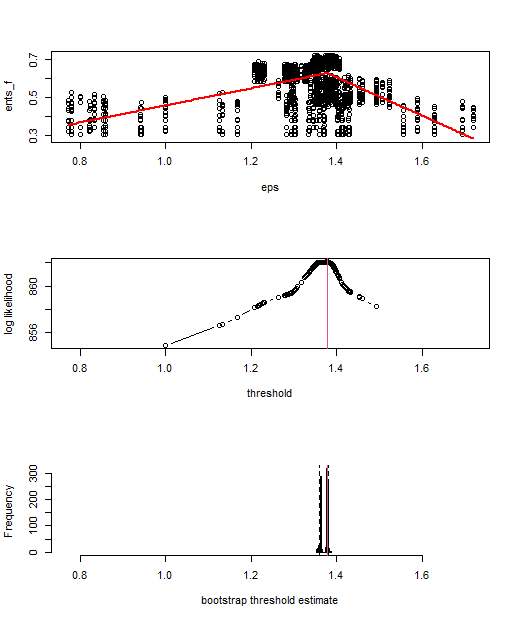
**

**Figure S8.** GPT-2 Threshold model for EPS
